# Association Between Dietary Zinc Intake and Metabolic Syndrome. A Meta-Analysis of Observational Studies

**DOI:** 10.3389/fnut.2022.825913

**Published:** 2022-02-03

**Authors:** Jun Ding, Qi Liu, Ze Liu, Hongbin Guo, Jieyu Liang, Yi Zhang

**Affiliations:** ^1^Changsha Social Work College, Changsha, China; ^2^Department of Orthopaedics, Xiangya Hospital, Central South University, Changsha, China; ^3^National Clinical Research Center for Geriatric Disorders, Xiangya Hospital, Central South University, Changsha, China

**Keywords:** dietary zinc intake, metabolic syndrome, meta-analysis, observational studies, clinical nutrition

## Abstract

**Background:**

Epidemiological studies have investigated the association between dietary zinc intake and metabolic syndrome (MetS). However, their results are conflicting. This meta-analysis was therefore employed to investigate the associations further.

**Methods:**

A comprehensive literature search was employed by using the electronic database of PubMed, Web of Science, and Embase up to November 2021. The pooled relative risk (RR) of MetS for the highest vs. lowest dietary zinc intake category, and the weighted mean difference (WMD) of dietary zinc intake for MetS vs. control subjects as well as their corresponding 95% confidence interval (CI) were calculated.

**Results:**

A total of 13 observational studies (18,073 participants) were identified in this meta-analysis. The overall multi-variable adjusted RR demonstrated that the dietary zinc intake was inversely associated with MetS (RR = 0.75, 95%CI: 0.61 to 0.93; *P* = 0.009). The subgroup analysis confirmed such findings in cross-sectional (RR = 0.70, 95%CI: 0.55 to 0.87; *P* = 0.002), NCEP-ATP III (RR = 0.64, 95%CI: 0.48 to 0.84; *P* = 0.002), adult (RR = 0.77, 95%CI: 0.62 to 0.96; *P* = 0.02), dietary recall method (RR = 0.70, 95%CI: 0.55 to 0.87; *P* = 0.002), and >500 sample-sized study (RR = 0.79, 95%CI: 0.64 to 0.99; *P* = 0.002), respectively. On the other hand, the overall combined WMD showed that the dietary zinc intake in MetS was also lower than that in control subjects (WMD = −0.21, 95%CI: −0.42 to 0.00; *P* = 0.05).

**Conclusions:**

Our results suggest that the dietary zinc intake is negatively associated with MetS. However, due to the limitation of available evidence. More well-designed prospective cohort studies are still needed.

## Introduction

Metabolic syndrome (MetS) is defined as a cluster of elevated fasting blood glucose, triglycerides, blood pressure, waist circumference, and decreased high-density lipoprotein cholesterol (at least three of the above metabolic abnormalities) (1). Metabolic syndrome is closely associated with diabetes mellitus, stroke and coronary heart disorders (2–4). The global prevalence of MetS is between 11.6 and 62.5%, which is still progressively growing (5). The etiology of MetS is not well-understood yet. However, the dietary factors are deemed to be significantly involved in MetS (6–10).

As the second most common trace metal in the body, zinc is associated with DNA replication and transcriptions, protein synthesis, and cellular division and differentiation (11). Zinc is an important antioxidant, which stabilizes membrane, prevents cellular apoptosis, and is also important for endothelial integrity (12, 13). It is widely accepted that zinc improves chronic inflammation, oxidative stress, and insulin resistance (14, 15), which is closely associated with the pathogenesis of MetS. Moreover, epidemiological data have indicated a negative relationship between dietary zinc intake and MetS-related context (e.g., diabetes) (16). Therefore, the dietary zinc intake is speculated to be inversely associated with MetS.

As far as we know, a number of observational studies have explored the association between dietary zinc intake and MetS (17–29). However, their results are still conflicting. Thus, this meta-analysis of observational studies is employed to investigate the issue further. It is hypothesized that the dietary zinc intake is inversely associated with MetS.

## Materials and Methods

### Search Strategy

Our meta-analysis was employed according to the Preferred Reporting Items for Systematic Reviews and Meta-analyses (PRISMA) guidelines (30). Combine the keywords that related to MetS (“metabolic syndrome”) and zinc (“zinc,” “zn”), the electronic database of PubMed, Web of Science, and Embase were searched up to November 2021. No language restriction was set in the search strategy. The titles and abstracts of all articles were screened firstly, and the full articles were then read to identify the eligible studies.

### Study Selection

The titles, abstracts and full texts of all retrieved studies were comprehensively reviewed by two researchers independently. Disagreements were resolved by discussions. The included studies were required to meet the following criteria: (1) the study design is observational study; (2) the association between dietary zinc intake and MetS; (3) the relative risk (RR), odds ratio (OR), or weighted mean difference (WMD) with 95% confidence interval (CI) were reported. The exclusion criteria were listed as follows: (1) duplicated or irrelevant articles; (2) randomized controlled trials; (3) reviews, letters, or case reports; (4) non-human studies.

### Data Extraction

The effect estimates from each included studies were extracted by two researchers independently, and disagreements were resolved by discussion. The information about the first author, year of publication, location, age, gender, sample size, study design, adjustments, dietary zinc assessment, exposure, effect estimates, and diagnostic criteria of MetS, was collected. The corresponding effect estimates of MetS for the highest vs. lowest dietary zinc intake category that adjusted for the maximum number of confounding variables were extracted for analysis. Moreover, the dietary zinc intake in MetS vs. control was also extracted to calculate the WMD (mean ± SD).

### Quality Assessment

We employed a quality assessment according to the Newcastle-Ottawa (NOS) criteria for non-randomized studies, which is based on three broad perspectives: the selection process of study cohorts, the comparability among different cohorts, and the identification of either the exposure or outcome of study cohorts. Disagreements were resolved by discussion.

### Statistical Analyses

The RR for MetS and WMD for dietary zinc intake were the outcome measures in the present study. The *I*^2^ statistic was employed to measure the heterogeneity by the percentage of total variation across studies (*I*^2^ > 50% was considered as heterogeneity). If significant heterogeneity was observed among the studies, the random-effects model was used; otherwise, the fixed effects model was accepted. Begg's test was employed to assess the publication bias (31). A *p*-value < 0.05 was considered as statistically significant. Moreover, a subgroup analysis was performed for study design, diagnostic criteria of MetS, population, exposure assessment, and sample size, respectively.

## Results

### Study Identification and Selection

**Figure 1:**
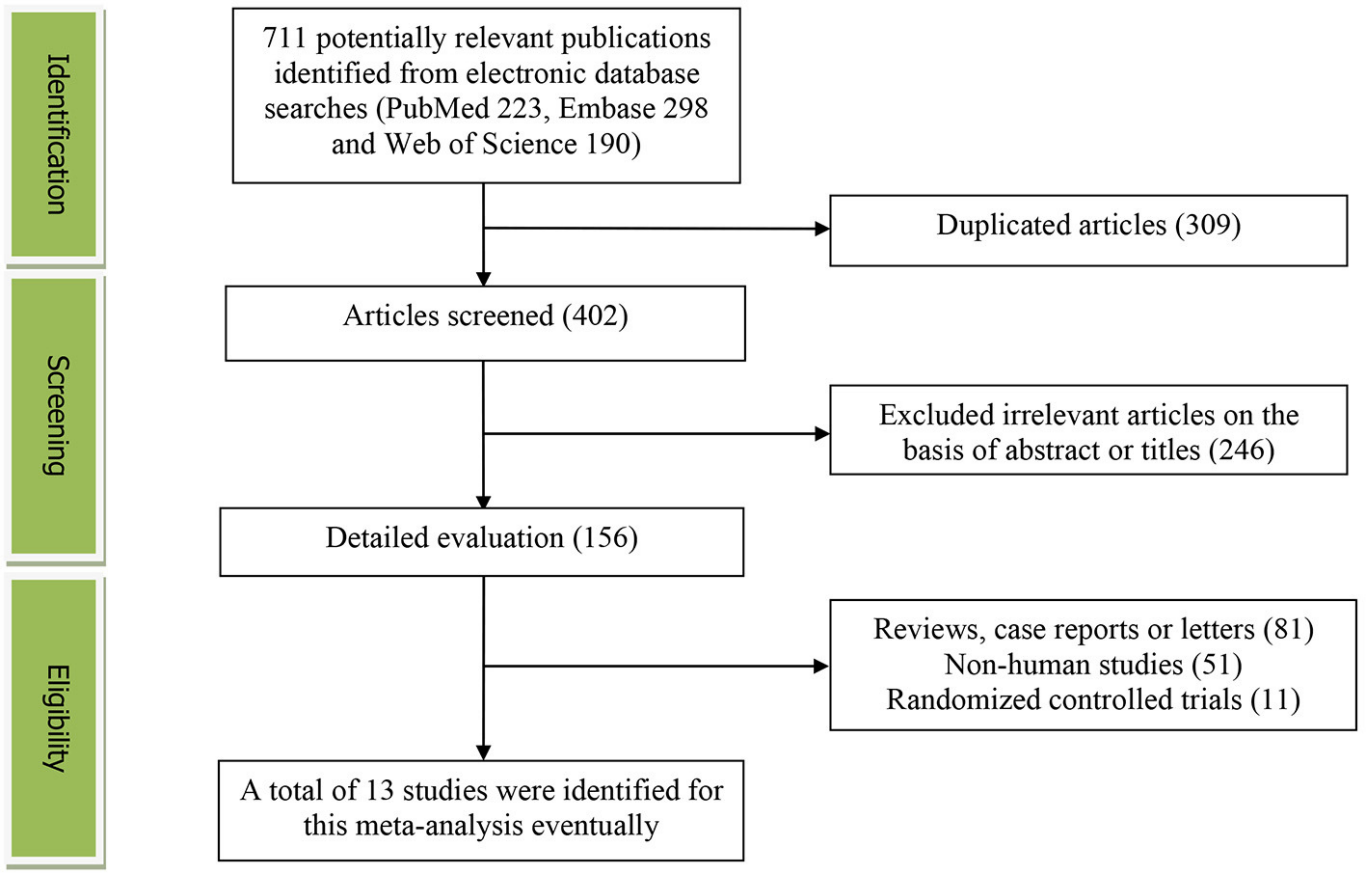
The detailed flow diagram of the study identification and selection in this meta-analysis.

Figure 1 presented the flow diagram of study identification and selection. Initially, a total of 711 articles (PubMed: 223, Embase: 298, and Web of Science: 190) were retrieved from the database during the literature search. After eliminating 309 duplicated articles, 402 articles were screened according to the titles and abstracts. Thereafter, 246 irrelevant studies were removed. Then, 81 reviews, case reports or letters, 51 non-human studies, 11 randomized controlled trials studies were excluded. Eventually, a total of 13 studies were identified for this meta-analysis.

### Study Characteristics

The main characteristics of the identified studies were presented in Table 1. These studies were published between 2008 and 2021. Seven of them were employed in Asian countries [China (22, 23, 27), Korea (17, 26), and Iran (24, 29)], and the other six ones were conducted in Brazil (18, 28), US (20), UK (21), Finland (19), and Columbia (25), respectively. Most studies considered both male and female participants, whereas Bruscato's study only recruited females (18). The sample size ranged from 42 to 5,323 for a total of 18,073. The dietary zinc intake was assessed by food-frequency questionnaire (FFQ) in three studies (17, 20, 29), and dietary recall method in 10 studies (18, 19, 21–28). The criteria for MetS were National Cholesterol Education Program-Adult Treatment Panel III (NCEP ATP III) (17, 19, 22, 23, 26, 27, 29), International Diabetes Federation (IDF) (18, 21, 24), and American Heart Association (AHA) (20) in 7, 3, and 1 studies, respectively. Moreover, the Ferranti's (32) and Cook's (33) criteria were employed for adolescent population (25, 28).

**Table 1:** Characteristics of the individual studies included in this meta-analysis.

**References**	**Location**	**Age years**	**Gender**	**Sample size**	**Study design**	**Adjustments**	**Dietary zinc assessment**	**Exposure**	**Effect estimates**	**Diagnostic criteria of MetS**	**NOS**
Kim (17)	Korea	Middle-aged	Both	688	Cross-sectional	NA	FFQ		Dietary zinc intake Male 5.50 (5.38, 5.62) 5.60 (5.46, 5.74) Female 5.80 (5.66, 5.94) 5.50 (5.36, 5.64)	NCEP-ATP III	6
								Control MetS Control MetS			
Bruscato (18)	Brazil	69.3 ± 6.3	Female	284	Cross-sectional	Age, smoking, years of education, physical activity, and dietary fiber	Dietary recall	Dietary zinc intake Quartiles 1 Quartiles 2 Quartiles 3 Quartiles 4 Control MetS	1.00 0.73 (0.36, 1.47) 0.54 (0.25, 1.13) 0,98 (0.47, 2.00) Dietary zinc intake 11.40 (10.65, 12.15) 11.00 (9.83, 12.17)	IDF	7
Kouki (19)	Finland	57–78	Both	1334	Cross-sectional	Age, alcohol consumption, smoking, education, and VO_2_max	Dietary recall	Dietary zinc intake Male Per mg/day Female Per mg/day Male Control MetS Female Control MetS		NCEP-ATP III	6
									0.97 (0.90, 1.06) 0.99 (0.94, 1.05) 5.50 (5.38, 5.62) 5.60 (5.46, 5.74) 5.80 (5.66, 5.94) 5.50 (5.36, 5.64)		
Otto (20)	US	45–84	Both	3828	Cohort	Energy intake, age, sex, race-ethnicity, education, study center, alcohol intake, physical activity, BMI, fiber intake, cigarette smoking, dietary supplement use the ratio of polyunsaturated fat intake: saturated fat intake and mutual adjustment for Mg, heme iron, non-heme iron, and antioxidant intake.	FFQ	Dietary zinc intake Quintiles 1 Quintiles 2 Quintiles 3 Quintiles 4 Quintiles 5	1.00 (0.78, 1.28) 1.20 (0.93, 1.55) 1.13 (0.85, 1.49) 1.33 (0.97, 1.82)	AHA	8
Al-Daghri (21)	UK	19–60	Both	185	Cross-sectional	Age, BMI, and physical activity	Dietary recall	Dietary zinc intake Quartiles 1 Quartiles 2 Quartiles 3 Quartiles 4 Control MetS	1.00 0.11 (0.04, 0.31) 0.17 (0.06, 0.50) 0.20 (0.07, 0.57) Dietary zinc intake 7.1 (6.5, 7.7) 6.1 (5.3, 6.6)	IDF	7
Bian (22)	China	30–70	Both	258	Cross-sectional	NA	Dietary recall	Control MetS	Dietary zinc intake 11.9 (11.5, 12.3) 12.5 (12.0, 13.0)	NCEP-ATP III	7
Li (23)	China	18–65	Both	550	Cross-sectional	Age, sex, and energy intake	Dietary recall	Dietary zinc intake Quartiles 1 Quartiles 2 Quartiles 3 Quartiles 4 Control MetS	1.00 0.33 (0.20–0.56) 0.34 (0.20–0.57) 0.18 (0.10–0.32) Dietary zinc intake 8.01 (7.64, 8.38) 7.22 (6.85, 7.59)	NCEP-ATP III	7
Motamed (24)	Iran	35–65	Both	3800	Cross-sectional	Sex, age, physical activity level, smoking, past medical history, energy intake, and BMI;	Dietary recall	Dietary zinc intake Quintiles 1 Quintiles 2 Quintiles 3 Quintiles 4 Quintiles 5 Male Control MetS Female Control MetS	1.00 1.06 (0.80, 1.30) 1.37 (1.09, 1.70) 1.19 (0.90, 1.40) 1.20 (0.97, 1.50) Dietary zinc intake 7.07 (6.87, 7.27) 7.02 (6.82, 7.22) Dietary zinc intake 6.98 (6.95, 7.01) 7.15 (7.03, 7.27)	IDF	8
Suarez (25)	Colombia	11–16	Both	1311	Cross-sectional	Age, BMI, socioeconomic status, and intakes of fat, carbohydrates, protein, and ascorbic acid	Dietary recall	Dietary zinc intake Male Tertiles 1 Tertiles 2 Tertiles 3 Female Tertiles 1 Tertiles 2 Tertiles 3		Ferranti's criteria	7
									1.00 NA 0.20 (0.05, 0.80) 1.00 NA 1.29 (0.56, 2.97)		
Lim (26)	Korea	52.5	Both	143	Cross-sectional	NA	Dietary recall	Control MetS	Dietary zinc intake 8.50 (7.89, 9.11) 8.10 (7.41, 8.79)	NCEP-ATP III	6
Zhu (27)	China	>18	Both	5323	Cross-sectional	Age, sex, region, years of education, physical activity level, intended physical exercises, smoking status, alcohol use, daily energy intake, iron, and magnesium	Dietary recall	Dietary zinc intake Quartiles 1 Quartiles 2 Quartiles 3 Quartiles 4	1.00 0.76 (0.63, 0.92) 0.55 (0.44, 0.69) 0.46 (0.35, 0.61)	NCEP-ATP III	7
Batista (28)	Brazil	<18	Both	327	Cross-sectional	Sex, age, maternal education, family income, physical activity, and alcohol intake	Dietary recall	Dietary zinc intake Tertiles 1 Tertiles 2 Tertiles 3	1.00 0.54 (0.21, 1.37) 0.46 (0.13, 1.63)	Cook's criteria	7
Zaeemzadeh (29)	Iran	18–40	Both	42	Case-control	NA	FFQ	Control MetS	Dietary zinc intake 10.46 (8.68, 12.24) 6.76 (3.05, 10.47)	NCEP-ATP III	5

### Relative Risk of MetS for the Highest vs. Lowest Dietary Zinc Intake Category

**Figure 2:**
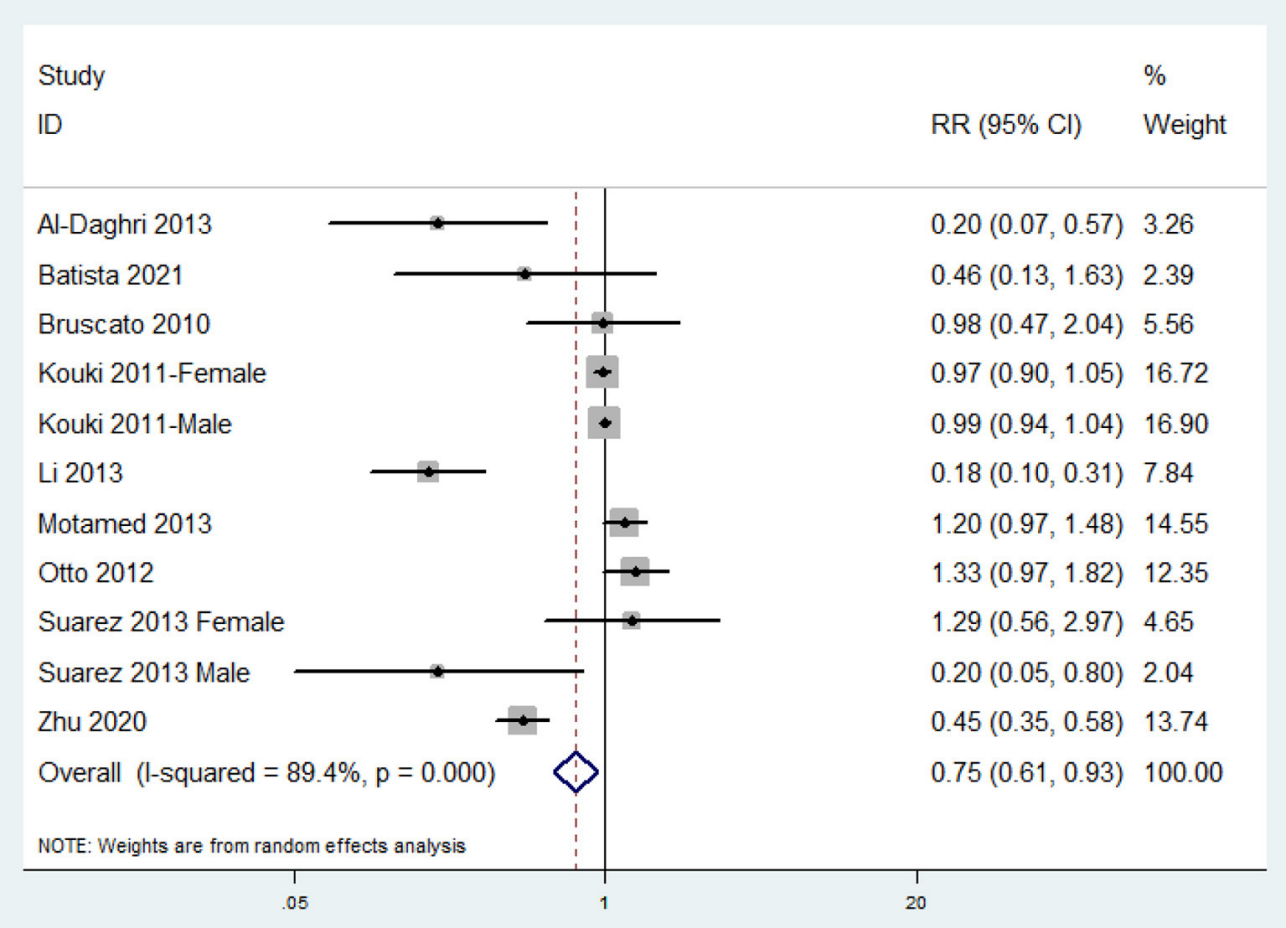
Forest plot of meta-analysis: Overall multi-variable adjusted RR of MetS for the highest vs. lowest dietary zinc intake category.

The overall multi-variable adjusted RR showed that the dietary zinc intake was inversely associated with MetS (RR = 0.75, 95%CI: 0.61 to 0.93; *P* = 0.009) (Figure 2). A substantial level of heterogeneity was observed among the various studies (*P* < 0.001, *I*^2^ = 89.4%). No evidence of publication bias was observed among the included studies according to Begg's rank-correlation test (*P* = 0.276). The results of subgroup analysis were presented in Table 2. Such findings were confirmed in cross-sectional (RR = 0.70, 95%CI: 0.55 to 0.87; *P* = 0.002), NCEP-ATP III (RR = 0.64, 95%CI: 0.48 to 0.84; *P* = 0.002), adult (RR = 0.77, 95%CI: 0.62 to 0.96; *P* = 0.02), dietary recall method (RR = 0.70, 95%CI: 0.55 to 0.87; *P* = 0.002), and >500 sample sized study (RR = 0.79, 95%CI: 0.64 to 0.99; *P* = 0.002), but not cohort (RR = 1.33, 95%CI: 0.97 to 1.82), other criteria of MetS (RR = 0.83, 95%CI: 0.55 to 1.26; *P* = 0.38), adolescent (RR = 0.55, 95%CI: 0.18 to 1.66; *P* = 0.29), FFQ (RR = 1.33, 95%CI: 0.97 to 1.82), and <500 sample sized (RR = 0.47, 95%CI: 0.17 to 1.29; *P* = 0.14) study.

**Table 2:** Subgroup analysis of MetS for the highest vs. lowest dietary zinc intake category.

**Stratification**	**Number of studies**	**Pooled RR**	**95% CI**	***P*-value**	**Heterogeneity**
All studies	9	0.75	0.61, 0.93	*P* = 0.009	*P* < 0.001; *I*^2^ = 89%
Study design					
Cross-sectional	8	0.70	0.55, 0.87	*P* = 0.002	*P* < 0.001; *I*^2^ = 90%
Cohort	1	1.33	0.97, 1.82	/	/
Diagnostic criteria of MetS					
NCEP-ATP III	3	0.64	0.48, 0.84	*P* = 0.002	*P* < 0.001; *I*^2^ = 96%
Other	6	0.83	0.55, 1.26	P = 0.38	*P* = 0.003; *I*^2^ = 70%
Population					
Adult	7	0.77	0.62, 0.96	*P* = 0.02	*P* < 0.001; *I*^2^ = 92%
Adolescent	2	0.55	0.18, 1.66	*P* = 0.29	*P* = 0.06; *I*^2^ = 64%
Exposure assessment					
FFQ	1	1.33	0.97, 1.82	/	/
Dietary recall method	8	0.70	0.55, 0.87	*P* = 0.002	*P* < 0.001; *I*^2^ = 90%
Sample size					
<500	3	0.47	0.17, 1.29	*P* = 0.14	*P* = 0.05; *I*^2^ = 67%
>500	6	0.79	0.64, 0.99	*P* = 0.04	*P* < 0.001; *I*^2^ = 92%

### Weighted Mean Difference of the Dietary Zinc Intake for MetS vs. Control Subjects

**Figure 3:**
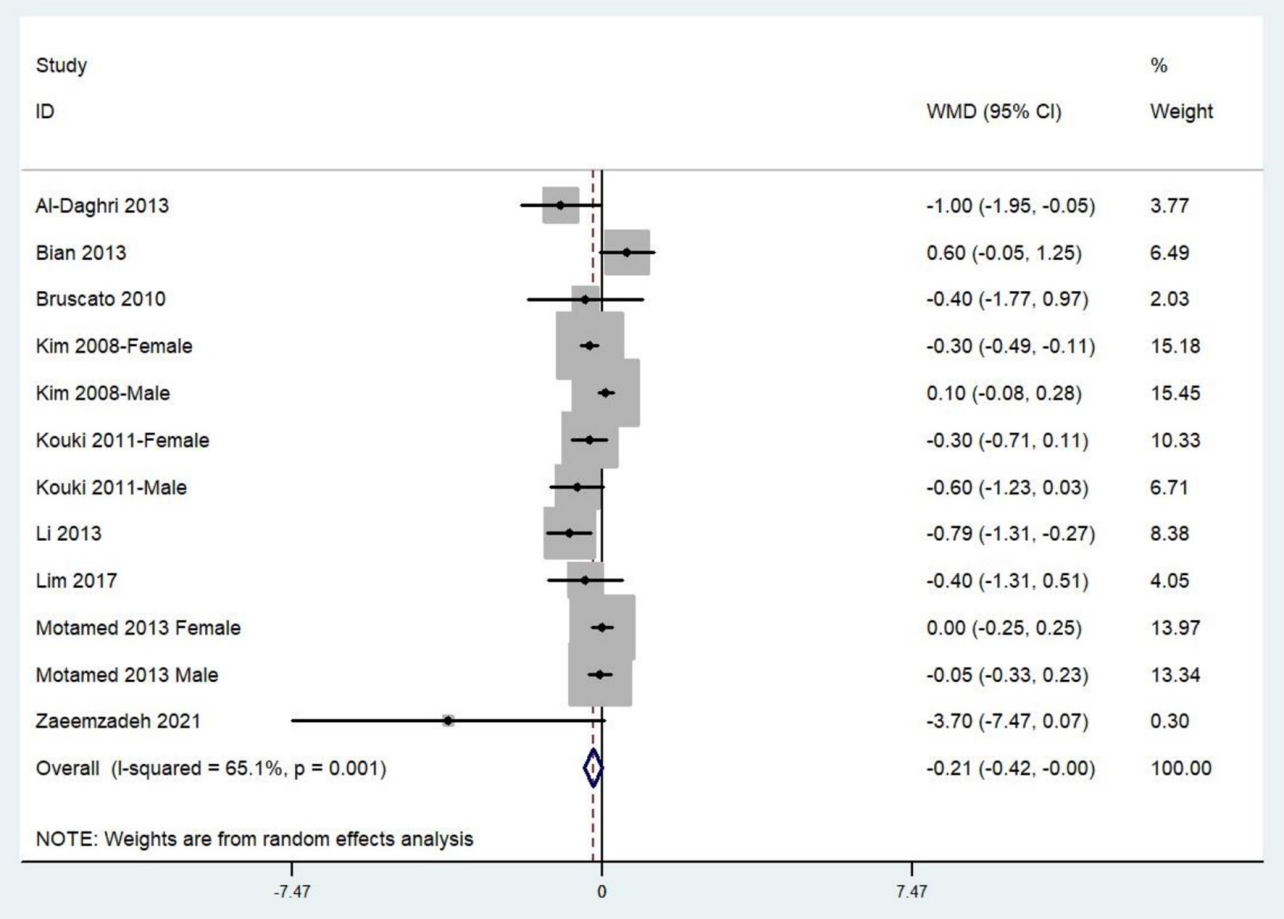
Forest plot of meta-analysis: Weighted mean difference of dietary zinc intake for MetS vs. control subjects.

The combined WMD demonstrated that the dietary zinc intake in MetS was lower than that in control subjects (WMD = −0.21, 95%CI: −0.42 to 0.00; *P* = 0.05) (Figure 3). A substantial level of heterogeneity was observed among the various studies (*P* = 0.001, *I*^2^ = 65.1%). No evidence of publication bias was observed according to Begg's rank-correlation test (*P* = 0.304).

## Discussion

In this study, a total of 13 observational studies are identified for meta-analysis. The results show that the dietary zinc intake is inversely associated with MetS. Moreover, the dietary zinc intake in MetS is lower than that in control either.

It is well known that both oxidative stress and inflammation plays significant role in the pathophysiology of MetS (34), and the antioxidant and anti-inflammatory property of zinc may mainly account for the negative relationship between dietary zinc intake and MetS. Consistently, several randomized controlled trials have revealed that zinc supplementation improves insulin resistance, oxidative stress, and inflammation in MetS subjects (35, 36). Moreover, zinc supplementation also leads to a higher level of TNF-α bound monocytes, which may benefit the immune response system (37). On the other hand, some fundamental experimental evidence indicates that long term zinc supplementation directly improves MetS in animal model (38), and decreases several metabolic disorder makers, lipid accumulation, and toxicity (39–41). Above all, the existing clinical and experimental data are strongly consistent with our results.

Interestingly, the inverse relationship between dietary zinc intake and MetS is only obtained in cross-sectional studies. Nevertheless, the number of cohort studies is rather small (only one), which may inevitably reduce the reliability. Moreover, the inconsistent result with regard to diagnostic criteria of MetS, exposure assessment and sample size is also acquired. It is speculated that NCEP ATP III criteria, dietary recall method, and lager sample size (>500) are more precise and suitable for this analysis. On the other hand, our findings only exist in adult, but not adolescent population. Indeed, the adolescent is a less concerned population for MetS (MetS is a chronic disorder, and only two studies are identified for adolescent). Our results preliminarily suggest a potential effect of age on the relationship between dietary zinc intake and MetS. Taken together, more well-designed prospective cohort study with the specification of population age (adult/adolescent) is still needed.

Several similar meta-analysis studies should also be noted. Capdor et al. find that zinc supplementation reduces glucose concentrations and HbA1c, which may contribute to the management of hyperglycemia in individuals with MetS (42). Moreover, Khazdouz et al. further indicates that zinc supplementation has beneficial effects on glycemic indices and lipid profile, which contributes to a reduction in risk of atherosclerosis (43). In addition, Karamali et al. demonstrates that 30 mg/day zinc supplementation for 6 weeks has beneficial effects on metabolic profiles in gestational diabetes subjects (44). These evidences strongly suggest a potential beneficial effect of zinc supplementation on MetS, which is a significant supplement for our results.

The relationship between serum zinc level and MetS has been deeply discussed in our previous work (45). It demonstrates that the serum zinc level in MetS is slightly higher than that in control, and an increased serum zinc level might be associated with a higher risk of MetS. However, these results seem to be limited by available evidence. More importantly, the development of MetS is associated with the chronic inflammation and oxidative stress (46–48), which lead to a lower serum zinc level. In turn, zinc can also reduce inflammatory cytokine production and oxidative stress (14, 45). As a consequence, the level of serum zinc might be dynamic in MetS condition. Alternatively, the dietary zinc intake is also served as a valid and reliable indicator for zinc status (49–53). Interestingly, a negative relationship between dietary zinc intake and MetS was obtained in our present study, which may encourage to build a collaboration between physicians and nutritionists to reinforce the dietary education in MetS subjects. Nevertheless, the toxicity of excess zinc intake should not be ignored neither. Excess zinc intake leads to the aggravation of renal function and an increase in systemic blood pressure predominantly through the oxidative stress (54). Moreover, excess dietary zinc intake may have negative impacts on epithelial signaling pathways, barrier function, and luminal ecology in the intestine, which may have long-term consequences on intestinal health (55). Therefore, a careful clinical validation is still needed before its application.

Our study has several strengths. First, this is the first meta-analysis of observational studies on the association between dietary zinc intake and MetS. Second, the included studies are analyzed based on the adjusted results and large samples. Third, our results may be beneficial for the nutritional management in MetS. The limitations of the present study should also be acknowledged. First, the reliability of our results might be influenced by the substantial level of heterogeneity. Second, due to the limitation in the relevant literature, only one prospective cohort study is identified (precludes causal relationships). Third, the classification of exposure varies greatly among individuals. Fourth, the selection of adjusted factors and definition of MetS are not uniform. Finally, only two studies have considered the adolescent population. These limitations may weaken the significance of this study.

## Conclusions

Our results suggest that the dietary zinc intake is negatively associated with MetS. However, due to the limited evidence, more well-designed prospective cohort study with the specification of population age is still needed to elaborate the issues examined in this study.

## Data Availability Statement

The original contributions presented in the study are included in the article/supplementary materials, further inquiries can be directed to the corresponding author.

## Author Contributions

YZ was the guarantor of the overall content, conceived the idea, and assessed each study. JD and YZ drafted this study. ZL and QL performed the statistical analysis. HG and JL selected and retrieved relevant papers. All authors revised and approved the final manuscript. All authors contributed to the article and approved the submitted version.

## Funding

This study was supported by National Natural Science Foundation of China (82102581), National Postdoctoral Science Foundation of China (2021M693562), Provincial Outstanding Postdoctoral Innovative Talents Program of Hunan (2021RC2020), Provincial Natural Science Foundation of Hunan (2019JJ40517), Young Investigator Grant of Xiangya Hospital, Central South University (2020Q14), and FuQing Postdoc Program of Xiangya Hospital, Central South University (176).

## Conflict of Interest

The authors declare that the research was conducted in the absence of any commercial or financial relationships that could be construed as a potential conflict of interest.

## Publisher's Note

All claims expressed in this article are solely those of the authors and do not necessarily represent those of their affiliated organizations, or those of the publisher, the editors and the reviewers. Any product that may be evaluated in this article, or claim that may be made by its manufacturer, is not guaranteed or endorsed by the publisher.
